# Comparison of Association Between Divorce and Access to Healthcare Services Among Married Immigrants

**DOI:** 10.1093/geroni/igab046.1984

**Published:** 2021-12-17

**Authors:** Suyeong Bae, James Graham, Sanghun Nam, Ickpyo Hong

**Affiliations:** 1 Yonsei University, Wonju, Kangwon-do, Republic of Korea; 2 Colorado State University, Colorado State University, Colorado, United States

## Abstract

The number of married immigrants is increasing in Korea, and family dissolution is also growing in this population. Although divorce could impact immigrants' health status, it is unclear whether they have difficulties accessing healthcare and medical services. Thus, we examined whether divorce in married immigrants is independently associated with access to healthcare services. A retrospective analysis of 11,778 adults who participated in the 2018 National Multicultural Family Survey. We used three different covariate adjustment methods (multivariate logistic regression, inverse probability of treatment weighting, 1:1 greedy propensity score matching) to examine the association between divorce and access to healthcare services after accounting for various demographic and clinical characteristics. Overall, 5.8% (n = 691) of married immigrants reported a history of divorce. The divorce group included 107 (15.5%) males and 584 (84.5%) females, with an average age of 45.17 years (SD = 10.9). The non-divorced group included 1992 males (18.0%) and 9095 (82.0%) females, with an average age of 39.1 years (SD = 10.5). After propensity score matching, all variables were balanced (all p>0.05). Individuals who experienced divorce were more likely to have difficulties in healthcare service access than those who did not experience divorce (adjusted odds ratio 1.423, 95% CI [1.075, 1.882]). Our findings revealed that divorce increased the risk of limited healthcare services among immigrants in Korea. Healthcare policymakers should be aware of the healthcare access issues in this minority population. In addition, to improve the lifestyles of minority populations, it is necessary to study their overall lives.

